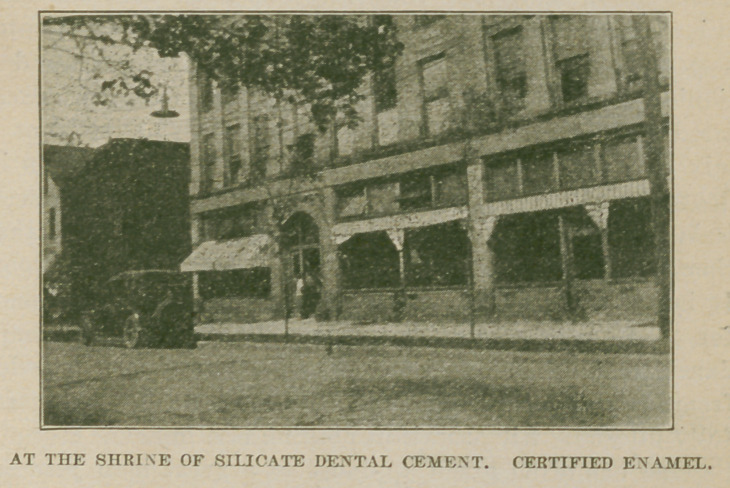# Event and Comment

**Published:** 1923-10

**Authors:** 


### DENTAL REGISTER

THE MAGAZINE OF DENTISTRY

Vol. LXXVII OCTOBER, 1923 No. 10

### EVENT AND COMMENT

BY VOTRE AMI DENTAIRE

### A LITTLE JOURNEY TO THE FACTORY OF A CELEBRATED DENTAL SILICATE CEMENT MANUFACTURER

Before releasing a "Little Journey," the original writer, who went down with the Lucitania, paragraphed and phrased the subject-matter as a sculptor molds his clay,

AT THE SHRINE OF SILICATE DENTAL CEMENT. CERTIFIED ENAMEL.

so that the result would be a cameo fit to adorn the walls of princely palaces and stately American homes. It is hoped that the reader will not judge this little journey with the degree of exactness to which the original Little Journey of Elbert Hubbard could be subjected. The

"Little Journey" described in this article does not deal with the artistic world, but with the practical.

We approached the cement factory in a rubber-tired tractor, onto which the government, along with other already existing laws, now proposes to place a twenty-mile speed regulator. This, by the way, is a little bit of Railroading. Why not make it impossible for them to run at less than twenty miles an hour so they will not compete with the street cars, which go nice and slow for shopping. Of course then, when you motor through the country you would have to go at a shopping speed, or when in town, park your automobile in the garage because it goes too fast for city driving. Monopoly of other forms of locomotion or speed thereof should not be given in traction or railroad franchises.

Cement in the dental sense is a tooth-filling material compared with cement in the concrete sense where it is a construction material. Naturally one must conclude that it is a hard substance and besides a very mixed one. Dental cement is a substance that results after a powder and a liquid of certain definite chemical proportions are spatulated to a "mix," not Tom, nor any fool "Mix," but a mix according to directions which every dentist learns in dental college. Aspinwall is the place where these chemicals are so minutely gauged and correctly assembled that they will "mix" in any climate on the face of the globe. It is not necessary to use even one synthetic ingredient, for the pure original chemicals are at Aspinwall in abundance, making it unnecessary to compound any powder from lesser ingredients. The liquid too is good, being carefully filtered. All American nothing else but.

One has often given applause to a great man just because many others do. Often applause starts because several are bold enough and seemingly appreciative enough to start it and the bowing of the performer works up a fanfare. But after one has become conversant with the subject through the helpful assistance of the master mind, over the tedious parts so to say, one can intelligently appreciate the obstacles that have been overcome, can understand the greatness of the achievement and then stand with hat in hand, uncovered head, in reverence acknowledging the greatness of the accomplishment. You then give your applause spontaneously because through your knowledge of the subject the achievement appeals to you. You find what others have already found. The researches of the Mellon Institute which are of inestimable importance have preceded the release of these Smith cements and Silicate enamels, the latter being called "Certified" because at the start one thousand of America's most prominent dentists, besides the chemists of the Mellon Institute, certified as to its perfection as a Silicate Cement, and as to its correctness in formula of uncombined unsynthesized elemental chemicals. It is nature's real Silicate like the precious diamond. Laboratory methods can only imitate and produce a substitute. That was where the band played "A Perfect Day" and ever since has played "Love Sends a Gift of Roses."

He who makes cements rides in automobiles because it is the sport of Dental Manufacturers. To and from the factory in a limousine makes it unnecessary to watch the weather. A speed regulator on motors in Pittsburgh would interfere very much with traction. Why build such smooth roads and deny one the use of them with ease at any greater speed than you could use the old roads with discomfort. That is analogous to asking why should a dentist deny his patient the safety from infection by not using Smith's Copper Cement in all cases rather than by using a copper cement only where indicated. Materials used makes a difference. A smooth road makes it safe at all speeds, so does Smith Copper Cement make it safe from previous infection or any subsequent infection that may arise.

A cement factory such as Mr. Smith's at Aspinwall is a busy place with shipments from its shipping department leaving every day. Large shipping and freight facilities pass within one square of the works. Mr. Linford Smith, the general manager, as he so often is called, or G. M., is so genial and genuine a man that everybody likes him. The policy of distribution of the Smith products is broad and generous. To those who have not been convinced of the fact that Smith's Certified Enamel is sticky, they must say it over again to themselves and realize that, if a Silicate Cement is not sticky how is it going to stand the test of adhesiveness. Unless a silicate is adhesive it will not answer the requirements of a silicate cement. Certified means that these features have been taken care of. Undercuts for retention are therefore not actually required, but only advised for added safety where tooth substance permits.

Our visit to the factory of the Lee S. Smith & Son Dental Mfg. Co., of Aspinwall on the Allegheny, near Pittsburg, leaves indeed a very happy recollection of the courtesy and welcome of its proprietor, Mr. W. Linford Smith, better known as Linford.

## Figures and Tables

**Figure f1:**